# Ocular Involvement in Infantile Cystinosis: A Case Report

**DOI:** 10.7759/cureus.70992

**Published:** 2024-10-07

**Authors:** Amine Razzak, Hala Ait Ammar, Mohamed Bouazza, Mohamed Elbelhadji

**Affiliations:** 1 Department of Ophthalmology, Cheikh Khalifa International University Hospital, Mohammed VI University of Sciences and Health, Casablanca, MAR; 2 Research Unit, Mohammed VI Center for Research and Innovation, Rabat, MAR

**Keywords:** conjunctival deposits, corneal deposits, cysteamine, cystinosis, infantile cystinosis

## Abstract

Infantile cystinosis is a rare systemic hereditary disorder characterized by abnormal cystine accumulation in cells, leading to various complications. Ophthalmological involvement is one of the major complications of this condition and significantly impacts visual prognosis. We report the case of a five-year-old male patient who was followed up for growth retardation, rickets, and refractory metabolic acidosis and was referred to ophthalmology for severe photophobia. Ophthalmological examination revealed a corrected visual acuity of 4/10 in the right eye and 8/10 in the left eye. Biomicroscopic examination showed birefringent corneal and conjunctival deposits. The diagnosis of infantile cystinosis was confirmed. Cystinosis is a lysosomal, autosomal recessive disease caused by intralysosomal cystine accumulation, manifesting ophthalmologically as cystine keratopathy and, less commonly, cystine retinopathy, which can threaten visual prognosis. The specific treatment for this condition is cysteamine, but management is multidisciplinary and must be initiated early to prevent severe complications.

## Introduction

Infantile cystinosis is a rare hereditary disease with autosomal recessive transmission, characterized by abnormal accumulation of cystine in tissues, leading to various complications. It is a metabolic disorder due to a lysosomal storage defect caused by mutations in the CTNS gene (cystinosin, lysosomal cystine transporter), which encodes the cystinosin protein responsible for transporting cystine out of the lysosomal compartment. The accumulation of cystine in the cells of multiple organs explains the systemic nature of this pathology. Renal involvement, growth retardation, endocrine disorders, and central nervous system involvement are the main extraocular manifestations of cystinosis. Ophthalmological involvement is generally detectable from the age of 12 months and constitutes the first extrarenal manifestation of cystinosis. It is mainly characterized by a pathognomonic corneal involvement that jeopardizes visual prognosis [1].

The diagnosis and therapeutic monitoring of the disease largely rely on measuring leukocyte cystine levels. The comprehensive management of cystinosis requires a multidisciplinary approach to prevent and treat the serious complications of the disease, which can affect the visual and vital prognosis. Notable progress has been made in recent years in treatment and monitoring, significantly improving prognosis. Additionally, the prescription of cysteamine both locally and systemically has delayed the onset of complications despite issues with tolerance and therapeutic adherence. We report the case of a five-year-old child with cystinosis, diagnosed based on polyuria-polydipsia syndrome and typical corneal involvement. This report highlights the clinical, diagnostic, and therapeutic aspects of this disease.

## Case presentation

The patient was a five-year-old male, born of a consanguineous marriage between first cousins, who had been followed up for growth retardation and severe metabolic acidosis. There was a family history of a similar case of cystinosis. He had been referred to an ophthalmology consultation for decreased visual acuity and photophobia.

**Figure 1:**
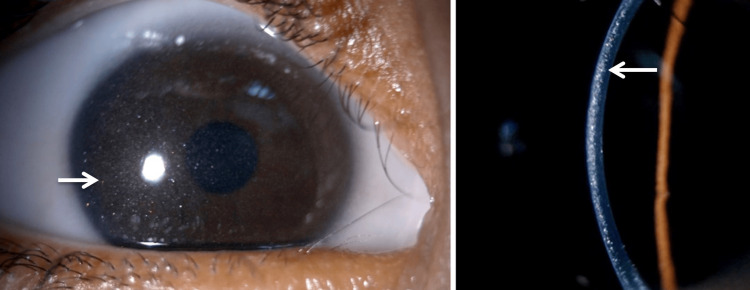
Slit-lamp photograph showing dense corneal cystine crystals White arrows: corneal deposits

**Figure 2:**
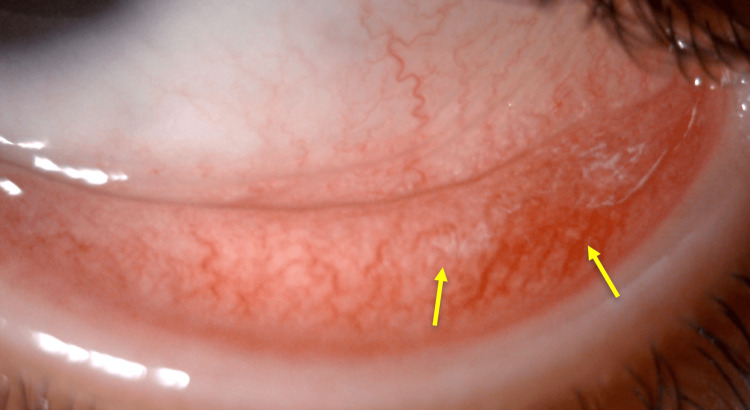
Slit-lamp photograph showing conjunctival cystine crystals Yellow arrows: conjunctival deposits

Clinical examination revealed corrected visual acuity of 4/10 in the right eye and 8/10 in the left eye. Intraocular pressure was normal in both eyes. Biomicroscopic examination revealed corneal deposits of birefringent crystals in both eyes with a density of 2.75 according to the Gahl score, without corneal erosion or neovascularization (Figure 1), associated with diffuse conjunctival deposits (Figure 2). Fundus examination did not reveal any signs of cystinosis retinopathy.

The diagnosis of infantile cystinosis was confirmed based on the pathognomonic appearance of corneal and conjunctival deposits and increased leukocyte cystine levels. The patient received symptomatic treatment for extraocular complications of the disease and was started on oral cysteamine (Cystagon®) at an initial dose of 800 mg per day, divided into four doses with regular monitoring. Topical treatment with cysteamine eye drops at a dosage of four drops per day was also initiated with good therapeutic compliance; a quarterly ophthalmologic follow-up was scheduled to evaluate the therapeutic response and ensure adherence to the treatment.

## Discussion

Cystinosis is an autosomal recessive monogenic hereditary disease caused by mutations in the CTNS gene located on the short arm of chromosome 17, encoding the protein cystinosin. Over 100 pathogenic mutations that can cause the infantile form have been reported in the literature [2], which results in a large variability that can influence both the severity and presentation of the disease, impacting early diagnosis, therapeutic response, and long-term management strategies. In our case, genetic analysis was not incorporated. Cystinosin transports cystine from the lysosome to the cytoplasm, where it is degraded into cysteine. Without this protein, intra-lysosomal accumulation of cystine occurs, manifested in the eye by the deposition of pathognomonic glittering corneal crystals detectable from the age of 12 months. These insoluble crystals can also deposit in various tissues: muscles, thyroid, pancreas, and central nervous system, leading to progressive multi-organ failure [3].

Aside from the infantile form, which is the most common clinical form (accounting for 95% of patients with cystinosis), the late-onset form is rare and may present with isolated corneal involvement [4]. Cystinosis nephropathy is the most common complication, as cystine accumulation in renal glomeruli causes severe and early proximal tubulopathy, which, without cysteamine treatment, progresses to end-stage renal disease [5]. Crystal deposits in various ocular structures explain the occurrence of variable ophthalmologic manifestations. The accumulation of cystine crystals in the cornea is the first extrarenal manifestation of cystinosis, initially presenting as high sensitivity to light or disabling photophobia. These crystals can be detected by slit-lamp examination from the age of one year and are constant from 18 months onwards [1]. Without local treatment, these deposits can lead to superficial punctate keratitis or even peripheral corneal neovascularization in older patients, ultimately resulting in corneal opacification [6].

Less commonly, cystinosis retinopathy is an ocular complication that can manifest from the age of six months onwards, characterized by peripheral retinal depigmentation with pigment epithelial mottling. This is one of the most dreaded ophthalmologic manifestations of cystinosis, as macular involvement leads to decreased visual acuity and can cause blindness in 10-15% of patients [7]. A thorough slit-lamp examination to detect characteristic corneal cystine crystals is one of the means of clinical confirmation. However, detecting elevated cystine levels in white blood cells remains the diagnostic method of choice. Additionally, CTNS gene molecular testing can be performed, detecting about 95% of the mutations responsible for the disease [6].

In our case, slit-lamp examination confirmed the diagnosis of cystinosis by revealing characteristic corneal and conjunctival deposits. However, the fundus examination did not show any retinal deposits. Early diagnosis of the disease improves prognosis if a multidisciplinary approach and regular follow-up are implemented, preventing and resolving severe complications. Aminothiol cysteamine (Cystagon®) is currently the only specific medication for cystinosis, reducing lysosomal cystine levels in all body tissues. Combined with symptomatic treatment for disease complications, cysteamine significantly improves overall prognosis by delaying progression to end-stage renal disease by 6-10 years, reducing the need for renal transplantation, especially in children [8]. While oral administration of cysteamine has no beneficial effects on corneal crystal deposits, local symptomatic treatment coupled with cysteamine eye drops or ophthalmic gel significantly reduces the density of corneal cystine crystals, improving symptoms, especially photophobia, despite a burning sensation associated with its acidic formulation, which may compromise therapeutic adherence [9].

In our case, symptomatic treatment and oral and topical administration of cysteamine resulted in general disease stabilization and ocular symptom improvement. However, long-term regular follow-up is necessary to evaluate therapeutic adherence and detect disease complications. Regular follow-up of patients with cystinosis is crucial to optimize management and prevent complications; this multidisciplinary follow-up should include ophthalmological consultations every three to six months to assess corneal crystal density and the emergence of other ocular complications, such as cystinosis retinopathy. Therapeutic adherence should be regularly checked, and cysteamine dosage adjustments may be necessary depending on disease progression and observed side effects.

## Conclusions

Close ophthalmologic monitoring is crucial in managing cystinosis, requiring thorough, meticulous, and repetitive clinical examinations to detect any early ophthalmic deterioration. Early diagnosis is vital to ensure better control of cystinosis, as early initiation of specific treatment allows for better growth and delays the onset of most renal and extrarenal complications. However, therapeutic adherence remains one of the major challenges in managing this condition. Cystinosis remains a severe disease requiring multidisciplinary clinical collaboration and social support for the patients and their families for effective treatment. Advances in the therapeutic landscape have altered the disease's course, enabling affected children to reach adulthood. Nevertheless, further research is needed to better understand the disease mechanisms and develop new therapeutic options.
